# Anthelminthic Activity of *Piper nigrum*, *Albizia ferruginea*, and *Guarea cedrata* Against *Caenorhabditis elegans* and *Heligmosomoides polygyrus*: In Vitro and In Silico Molecular Docking Approach

**DOI:** 10.1155/sci5/1600933

**Published:** 2025-12-02

**Authors:** Noumedem Anangmo Christelle Nadia, Yamssi Cédric, Djam Chefor Alain, Ndongmo Donjio Corine Leader, Masoud Besati, Mahdi Yaghoobi, Wenjuan Liu, Ye Liu, Haibo Hu

**Affiliations:** ^1^Department of Microbiology, Haematology and Immunology Faculty of Medicine and Pharmaceutical Sciences, University of Dschang, Dschang, Cameroon; ^2^Laboratory of Tropical and Emerging Infectious Diseases, Dschang, Cameroon; ^3^Department of Biomedical Sciences, Faculty of Health Sciences, University of Bamenda, Bambili, Cameroon; ^4^Department of Public Health, Faculty of Medicine and Pharmaceutical Sciences, University of Dschang, Dschang, Cameroon; ^5^Institute for Integrative Systems Biology (I2SysBio), CSIC−University of Valencia, Paterna 46980, Spain; ^6^Molecular Design and Synthesis, Department of Chemistry, KU Leuven, Leuven, B-3001, Belgium; ^7^Jiangxi Province Key Laboratory of Pharmacology of Traditional Chinese Medicine, National Engineering Research Center for Modernization of Traditional Chinese Medicine-Hakka Medical Resources Branch, School of Pharmacy, Gannan Medical University, Ganzhou 341000, China

**Keywords:** *Albizia ferruginea*, anthelmintics, *Caenorhabditis elegans*, *Guarea cedrata*, *Heligmosomoides polygyrus*, *Piper nigrum*

## Abstract

**Background:**

Gastrointestinal parasites, being members of the neglected tropical diseases (NTDs), infect over one billion individuals, about 24% of the global population. The aim of this study was to evaluate the deworming potential of *Piper nigrum, Albizia ferruginea*, and *Guarea cedrata* against *Caenorhabditis elegans* and *Heligmosomoides polygyrus* and to recommend their use in traditional medicine for the treatment of helminth infections.

**Methods:**

The anthelmintic properties of the extracts were investigated in two nematode strains, *Heligmosomoides polygyrus* and *Caenorhabditis elegans.* The fresh coprocultured *H. polygyrus* L3 larvae and *C. elegans* L4 larvae bleached from adult worms were used to investigate the properties. Larval movement was monitored using a worm microtracker in a 96-well microplate to quantify the anthelmintic action of the extracts. The extracts were screened at varying concentrations, with distilled water being the negative control and albendazole being the positive control. Percent inhibition of larval motility was calculated. Molecular docking studies were also carried out using the Glide module of Schrodinger Maestro software, and the results ranked and distinguished based on the software's scoring function.

**Results:**

The most active extract against *H. polygyrus* was the ethanolic extract of *Piper nigrum* (IC_50_:0.04 mg/mL) followed by the aqueous extract of *Piper nigrum* (IC_50_:0.08 mg/mL). Aqueous and ethanoic extracts of *Piper nigrum* were active against *Caenorhabditis elegans* L4 larvae with IC_50_s of 7.850 and 16.17 µg/mL, respectively, while aqueous extracts of *Guarea cedrata* and *Albizia ferruginea* were highly active with IC_50_s of 3.235 and 4.729 μg/mL, respectively. Leucokinin III, Leucokinin I, Leucokinin VIII, Leucokinin II, and Rebaudioside C from *Albizia ferruginea* are the most potent compounds against succinate dehydrogenase (SDH) and β-tubulin. Each of these constituents exhibited a more pronounced effect compared to the positive control, albendazole. Tricholein, isopiperolein B, pipercyclobutanamide, piperettine, and piperine from *Piper nigrum* are the most potent compounds against SDH and β-tubulin.

**Conclusion:**

This study has demonstrated in vitro and in silico the effectiveness of *Piper nigrum, Albizia ferruginea,* and *Guarea cedrata* toward helminthiasis. To validate this scientific investigation, more research is required, particularly on the acute toxicity and in vivo anthelmintic efficacy.

## 1. Introduction

Gastrointestinal parasites are a public health problem in developing countries (DCs), where climatic, hygienic, and demographic conditions favor the proliferation and transmission of gastrointestinal parasites [1]. These parasitoses threaten the socioeconomic development of DCs, with very high morbidity and mortality rates, causing enormous consequences on the medical level through the disorders they cause in parasitized subjects on the one hand and on the economic level through the costly therapeutic and preventive measures they impose [2]. According to estimates by the World Health Organization (WHO), 3.5 billion people are infected by digestive parasites, with an estimated morbidity of 450 million and a mortality rate of 155,000 cases per year [2]. Amebiasis, ascariasis, hookworm, and trichocephaliasis are among the 10 most frequent infestations worldwide, causing 195,000 deaths alone [3]. In fact, parasitic infections are closely linked to the absence of or poor sanitation (unavailability of drinking water, inadequate disposal of human waste, lack of latrines) or to poor personal hygiene [4]. These factors, which contribute to the endemicity and perpetuation of transmission, remain highly diverse and complex. To combat helminthiasis, populations and breeders use numerous synthetic products [5]. The first cases of drug-resistant gastrointestinal nematodes were reported as early as the 1960s in Australia, New Zealand, and South Africa [6]. Today, this phenomenon is known worldwide. In addition, drug shortages are frequent, and the cost of medicines is increasingly high [7]. Added to this is the noncompliance with prescribed doses to prescribed doses and the use of counterfeit products [8]. Medicinal plant preparations have been used by mankind since the dawn of time. In 2004, the WHO estimated that nearly 80% of the population in DCs relied on traditional pharmacopoeia as their main means of treatment [5]. Treatment with plants is favored by their availability, simple preparation, and low adverse effects [9, 10]. However, traditional pharmacopoeia has the disadvantage of being practiced empirically. In Cameroon, some local people use *Albizia ferruginea*, a plant belonging to the Fabaceae family, where the leaves are macerated and used to purge children and treat intestinal disorders [6].

*Piper nigrum* has a well-documented history in traditional medicine for treating intestinal worms and digestive disorders [11–13]. Piperine, its major alkaloid, has been reported to exhibit broad-spectrum anthelmintic and anti-inflammatory activities [14, 15]. The genus *Albizia* is traditionally used in ethnomedicine for the treatment of intestinal parasites, diarrhea, and fevers [16, 17]. Previous studies have shown the anthelminthics of *Albizia* [18–20].

*Guarea cedrata* is used to treat stomachaches and food poisoning, and *Piper nigrum*, whose seeds are commonly known as white pepper, is referred to as the king of spices due to its purifying capacity and in traditional medicine as an anti-inflammatory, anti-diabetic, and digestive aid [21]. Phytochemical screenings have identified limonoids and sesquiterpenes in this plant, compounds known for antiparasitic and cytotoxic activity [22]. The compounds used in this research are constituents extracted from different parts of *Piper nigrum* [23–26], *Albizia ferruginea* [27–29], and *Guarea cedrata* [30] and have been published in various articles in recent years.

The present study aimed to evaluate the anthelminthic activity of extracts from *Piper nigrum*, *Albizia ferruginea*, and *Guarea cedrata* against *Caenorhabditis elegans* and *Heligmosomoides polygyrus*, both in vitro and through in silico molecular docking. The in vitro assays were conducted to determine the efficacy of the extracts, while the in silico approach was used to understand the molecular interactions between the active compounds and the target receptors.

## 2. Materials and Methods

### 2.1. Plant Material and Identification of Plant Samples

The leaves of *Albizia ferruginea, Guarea cedrata*, and the seeds of *Piper nigrum* were harvested in the town of Dschang (Menoua Division, Western Region). *Piper nigrum* seeds were purchased at Dschang's B Market, while the leaves were harvested in fields. The plant species were identified at the Yaoundé's National Herbarium where voucher specimens have been deposited using the following reference numbers: *P. nigrum* (25818/SFR/CM), 49871/HNC for A. *ferruginea*. and *G. cedrata* (1378/SFR/CM).

### 2.2. Drying Plants

The *Piper nigrum* seeds were dried in the laboratory before being crushed. Once in the laboratory, the leaves were cut into small pieces and dried at room temperature. The leaves were then stirred regularly to prevent the development of mold. After drying, they were crushed using a blender. The resulting powders were used to prepare the various extracts.

### 2.3. Preparation of Ethanolic Extract

*Piper nigrum* seed powder ethanolic extract was prepared using the method outlined by Christelle et al. [31]. Two hundred grams of powdered seeds were weighed using a Mettler TOLEDO electric balance and added to 2 L of 95% ethanol. The contents were shaken for 5 minutes and left to infuse under seal at room temperature for 72 h. After maceration, the solution was filtered twice first using a 150 μm mesh sieve, and then using Whatman filter paper. The resulting filtrate was dried in an oven at 40°C.

### 2.4. Preparation of Aqueous Extract

The aqueous extract was obtained by infusing the dried leaf powder of the other plants. Briefly, 200 g of leaf powder were added to 2 L of distilled water heated at 100°C. The mixture was left to cool down for 3 h at room temperature. The filtrate was filtered twice as described above, and the filtrate obtained was dried at 40°C.

### 2.5. Animal Material

Two nematodes, the parasitic *Heligmosomoides polygyrus* and the free-living, genetically engineered model strain *Caenorhabditis elegans* (Bristol N2), were adopted. *Caenorhabditis elegans* is routinely used as a model for finding anthelmintic drugs due to its genetic and physiological equivalence to parasitic nematodes, ease of laboratory culture, and a brief life cycle. It allows rapid, low-cost, and ethically acceptable high-throughput screening of the drug candidates. It has a transparent body with which one can directly observe drug effects, and its neuromuscular system is well described and thus extremely appropriate for the investigation of mechanisms of action of neuroactive compounds. Also, the majority of anthelmintics known possess similar activity in *C. elegans* and therefore is a good indicator of antiparasitic activity. The intestinal parasite *Heligmosomoides polygyrus* is naturally present in mice and serves as a suitable model for gastrointestinal helminth infections in both humans and animals. This trichostrongyloid nematode parasite is commonly used by research facilities to assess the efficacy of anthelmintics.

### 2.6. Preparation of *H. polygyrus* L3 Larvae

The L3 larvae were obtained after coproculture in the laboratory using the method described by Cedric et al. [32]. Briefly, 5 g of fresh feces from infected Swiss mice was first collected and then crushed in a mortar using a pestle. Charcoal powder was then added in the ratio 1:1. Whatman paper was then delicately placed on a Petri dish and lightly soaked in distilled water. A small portion of the charcoal–feces mixture was then taken and spread on this Whatman paper in the Petri dishes. These Petri dishes were then placed in a jar and incubated at 20°C for 7 days. After 7 days, the Whatman paper supports on which the culture was performed were washed, and a solution containing L_3_ larvae was obtained.

### 2.7. Preparation of *C. elegans* L4 Larvae

The wild-type Bristol strain of *Caenorhabditis elegans* was maintained as per the protocol described by Liu et al. [33]. NGM was employed to culture the worms, while *Escherichia coli* strain OP50 was utilized as food. Synchronized eggs were cultivated at 20°C in S basal solution until hatching. The thus-preserved L1 larvae were later transferred to NGM and incubated for another 36 h to reach the L4 stage. The resulting L4 larvae were subsequently used for the anthelmintic assay.

### 2.8. Anthelmintic Activity on L3 Larvae of *H. polygyrus* and L4 Larvae of *C. elegans*

The anthelmintic activity was assessed using the method outlined by Cedric et al. [32]. In brief, a Worm Microtracker was employed to monitor larvicidal effects. Fifty L3 *H. polygyrus* larvae were treated with extracts at different concentrations (0.078–2.5 mg/mL) in a 96-microplate experiment. Higher concentrations are needed to evaluate the anthelminthic activities of compounds on *H. polygyrus* because there are more resistant than other nematodes such as *C. elegans* which require lower concentrations to kill them. The larvicidal activity against L_4_*C. elegans* larvae was tested following the identical protocol, using extract concentrations ranging from 6.25 to 200 μg/mL. Plates were incubated for 18 h at 20°C in the Worm Microtracker, where larval motility was continuously observed. In this study, albendazole (10 μg/mL) was used as the positive control for assays involving *Heligmosomoides polygyrus*, due to its well-established efficacy against gastrointestinal nematodes. For *Caenorhabditis elegans*, Levamisole (5 μg/mL) was employed as the positive control, given its known neuro-muscular inhibitory effects in this model organism and its standard use in anthelmintic screenings. All experiments were performed in triplicate. Worm motility was measured in each well every 30 min using an infrared microbeam that scanned each well more than 10 times per second using the Worm Microtracker. Anthelmintic activity was then assayed based on these measurements of motility.

(1) $$\begin{array}{c} \%\text{ Inhibition}=\frac{\text{Mobility activity of Control}-\text{Mobility activity of the test sample}}{\text{Mobility activity of Control}}\times 100. \end{array}$$

### 2.9. Molecular Docking

**Figure 1:**
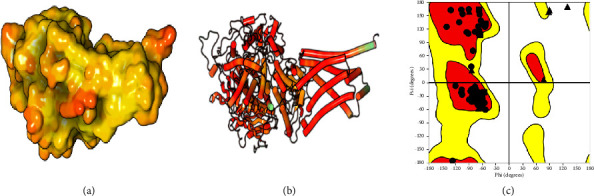
PDB ID: 4YSX. (a) The surface mode of the SDH model [19]; (b) optimized SDH model receptor [19]; and (c) Ramachandran plot of SDH model receptor.

Succinate dehydrogenase (SDH) was selected for the receptor of this evaluation as SDH inhibitors are highly effective against different types of the nematodes, including *Caenorhabditis elegans* [34]. Furthermore, some quinoline and triazole derivatives were reported as possible SDH inhibitors [35, 36]. The experimental crystal structure of *C. elegans* SDH is not available in the Protein Data Bank; hence, its 3D structure was modeled using the Swiss-model web server. The *C. elegans* sequences of the subunits (UniProt entry Q09545) were retrieved from the UniProt database (uniprot.org). The most relevant generated SDH structure was selected based on the Global Model Quality Estimation (GMQE) and Qualitative Model Energy Analysis (QMEAN) values. SDH of *Ascaris suum* (PDB ID: 4YSX: sequence identity with SDH of *C. elegans*—83.87%) was used based on homology modeling (Figure 1). Owing to the absence of a co-crystallized ligand with the β-tubulin chain and the SDH model obtained, we used the Sitemap module of Schrodinger for the detection of potential cavities for inhibitor binding. As a reference compound for estimating the inhibition potency of tubulin polymerization, we used albendazole, i.e., a popular and commercially successful drug, which binds to the same pocket of tubulin [37]. Therefore, the structure of tubulin was cleaned from water molecules and other entities (ions, cofactors, ligands). Polar hydrogens were added, nonpolar hydrogens were merged, and histidine protonation was modified manually. Kollman charges were calculated for the whole enzyme structure and spread over the residues. 3D structures of the compounds were made for further simulation [38].

The compounds used in this research are constituents extracted from different parts of *Piper nigrum* [23–26], *Albizia ferruginea* [27–29], and *Guarea cedrata* [30] and have been published in various articles in recent years. Here, through in silico screening, we attempted to explore molecular interaction of these ligands with nematode proteins for mechanistic understanding for the control measures. 3D models of all of the reference ligands for the SDH and no target were retrieved from PubChem database (https://pubchem.ncbi.nlm.nih.gov). The SDH grid boxes were created by the Receptor Grid Generation function of Maestro's Glide module. Two grids (10 × 10 × 10 and 20 × 20 × 20) were generated to define the enzyme active site, and the grid center was defined as X: 93.95, Y: 21.69, Z: 64.01 and selected ligand: 5CUESN4500.

Docking calculations were conducted using the Glide module of the Schrodinger Maestro software package [39]. The resultant ligand–protein adducts were evaluated and ranked against Glide's scoring function, then grouped by scores [40] (see Tables 1 and 2). These analyses allow predictions of three-dimensional complex structures from binding affinities and relative spatial orientation of ligands and target proteins. Docking predicts the likely ligand conformation and position within a specific binding site. Preprocessing of the protein structure was achieved through the “Protein Preparation Wizard” in Maestro to automatically create protonation states and optimize steps to add missing atoms, such as hydrogen and necessary bonds. Following optimization, receptor grid generation was performed, and docking scores for various ligand conformations were computed and compared [41–43].

### 2.10. Ethical Consideration

All authors hereby declare that the “Principles for the care of Laboratory Animals” (NIH Publication No. 85-23, revised 1985) have been followed, as well as specific national laws, where applicable. All experiments were reviewed and approved by the Institutional Ethics Committee for Research on Humans Health of the University of Douala under the registration number 057IEC-UD/11/2022/T.

### 2.11. Data Analysis

Data were initially processed using Microsoft Excel 16.0 to calculate the percentage inhibition, reduction, and suppression. Values were thereafter exported to GraphPad Prism version 8.4 to calculate IC_50_ values by plotting concentration–response curves, in which logarithmic concentration was plotted against percentage inhibition. Means comparison between groups was carried out using ANOVA, in which significance was at *p* < 0.05. Docking analyses were carried out using the Glide module of the Schrodinger Maestro software, where the scoring function was used for ranking and sorting the most likely ligand–protein adduct structures generated through molecular docking.

## 3. Results

### 3.1. Effects of Plant Extracts on *H. polygyrus L*3 Larvae

The anthelmintic activities of plant extracts on *H. polygyrus L*3 larvae are presented in Table 3. The most active extract against *H. polygyrus* was the ethanolic extract of *Piper nigrum* (IC_50_: 0.04) followed by the aqueous extract of *Piper nigrum* (IC_50_: 0.08). Albendazole (400 mg) was used as a positive control, while distilled water was used as a negative control.

### 3.2. Effects of Plant Extracts on *C. elegans* L4 Larvae

Table 4 shows the effect of the anthelminthic activity of plant extracts on *C. elegans* L4 larvae. Levamisole was used as a positive control in this case, and distilled water as a negative control. Aqueous and ethanolic extracts of *Piper nigrum* were active with IC_50_s of 7.850 and 16.17 μg/mL, respectively, while aqueous extracts of *Guarea cedrata* and *Albizia ferruginea* were highly active with IC_50_s of 3.235 and 4.729 μg/mL, respectively.

### 3.3. Analysis of Molecular Docking and In Silico Approaches for Anthelminthics

Using the Glide module, molecular docking between ligands and target proteins was performed [44, 45]. Several ligands demonstrated significant docking scores when they interacted with amino acids in target proteins. An overview of docking scores for the five leading ligands is provided in Tables 1 and 2. HTVS, SP, and XP molecular docking methodologies were used to screen compounds from *Piper nigrum, Albizia ferruginea,* and *Guarea cedrata.* A sample of 15% of the most stable ligands was screened in every step based on their docking scores. The most stable structures of ligands were docked using the XP docking score.

**Figure 2:**
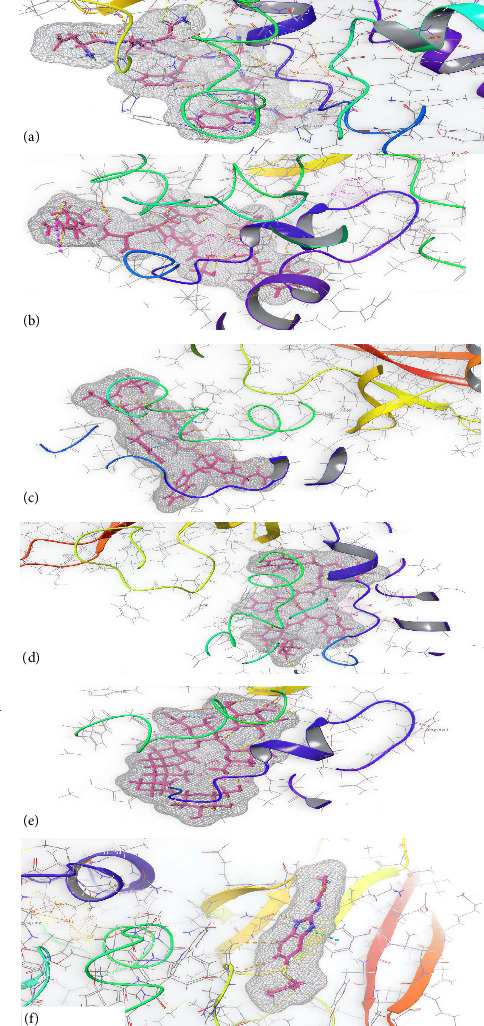
Three-dimensional (3D) interactions between the SDH and the ligands of *A. ferruginea*: (a) leucokinin III, (b) leucokinin I, (c) leucokinin VIII, (d) leucokinin II, (e) rebaudioside C, and (f) albendazole.

The in silico outcomes of *Albizia ferruginea* compounds are presented in Table 1 and Figure 2. From the figure, it was reported that Leucokinin III, Leucokinin I, Leucokinin VIII, Leucokinin II, and Rebaudioside C were the best inhibitors of SDH and β-tubulin. The compounds inhibited more strongly than the reference drug albendazole.

**Figure 3:**
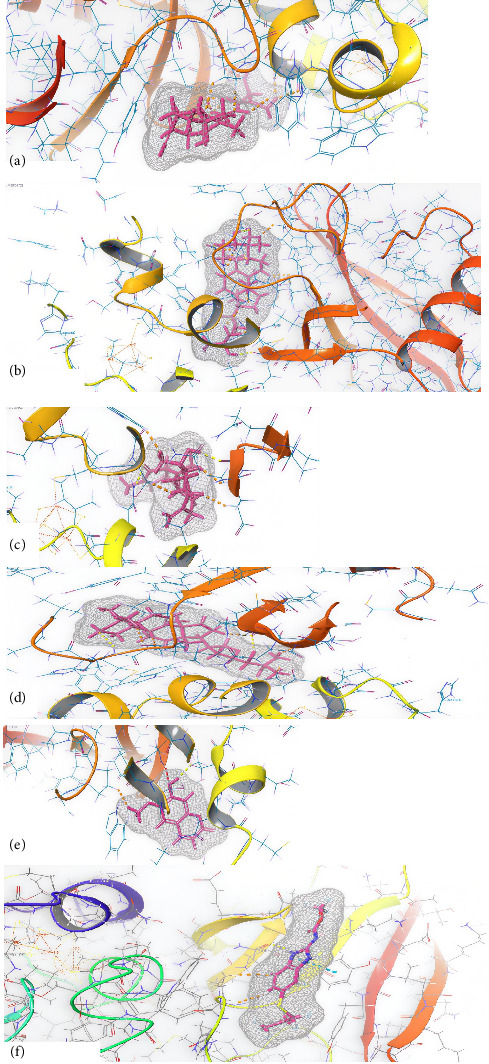
Three-dimensional (3D) interactions between the SDH and the ligands of *G. cedrata*: (a) boscartol A, (b) cycloart-23-ene-3beta,25-diol, (c) boscartol G, (d) melianodiol, (e) Scopoletin, and (f) albendazole.

Similarly, results for *Guarea cedrata* compounds are illustrated in Table 2 and Figure 3. Boscartol A, Cycloart-23-ene-3β,25-diol, Boscartol G, Melianodiol, and Scopoletin were found to score the maximum docking against SDH and β-tubulin. All of these constituents also ranked above albendazole in terms of forecasted binding affinity.

**Figure 4:**
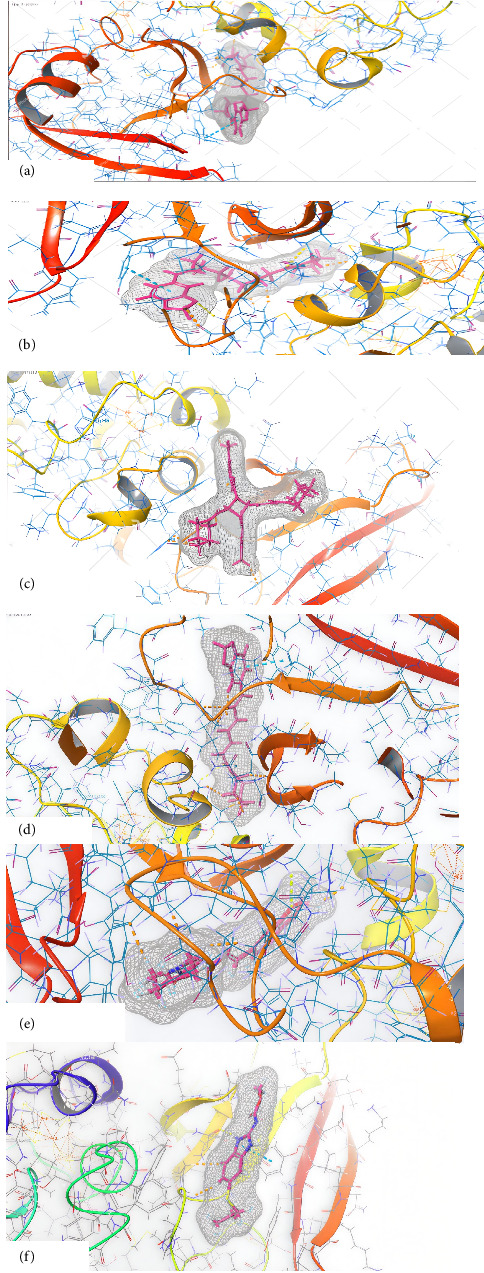
Three-dimensional (3D) interactions between the SDH and the ligands of *P. nigrum*: (a) tricholein, (b) isopiperolein B, (c) pipercyclobutanamide, (d) piperettine, (e) piperine, and (f) albendazole.

The results related to the in silico studies of the *Piper nigrum* compounds are reported in Table 5 and Figure 4: As illustrated in Table 5 and Figure 4, tricholein, isopiperolein B, pipercyclobutanamide, piperettine, and piperine from *Piper nigrum* are the most potent compounds against SDH and β-tubulin. Each of these constituents like another calculated constituents in this research exhibited a more pronounced effect compared to the positive control, albendazole.

## 4. Discussion

The IC_50_ values obtained in our study indicated that the L4 larvae of *C. elegans* were most sensitive to the aqueous extract of *Guarea cedrata* (IC_50_ = 3.235 μg/mL), followed by *Albizia ferruginea* (IC_50_ = 4.729 μg/mL). This demonstrates the strong larvicidal activity of both extracts. In a related study, Amazonas et al. [46] investigated the chemical profile and larvicidal activity of essential oils from four *Guarea* species against *Aedes aegypti* larvae. Their findings reinforced the efficacy of plant-derived compounds in preventing larval development. At higher concentrations (500 and 250 μg/mL), all essential oils exhibited 100% mortality after 24 h of exposure. The most active oils were obtained from *Guarea humaitensis* branches (LC_50_ = 48.6 μg/mL), *G. scabra* leaves (LC_50_ = 98.6 μg/mL), and *G. silvatica* leaves (LC_50_ = 117.9 μg/mL), which is in line with the anthelmintic activity observed in our study.

Our results are consistent with those of Tagoe et al. [18], in which *A. ferruginea* exhibited dose-dependent anthelmintic activity against *Pheretima posthuma* and *Haemonchus contortus*. The lowest concentration of *A. ferruginea* (0.5 mg/mL) was able to paralyze and kill *P. posthuma* within 272.50 ± 12.42 min and 354.50 ± 5.06 min of exposure, respectively.

The most active extract against *H. polygyrus* in our study was the ethanolic extract of *Piper nigrum* (IC_50_ = 0.04 μg/mL), followed by its aqueous extract (IC_50_ = 0.08 μg/mL). Similarly, Mohamed et al. [47] demonstrated the ovicidal, larvicidal, and adulticidal activity of black pepper (*Piper nigrum* L.) essential oil and tea tree oil (*Melaleuca alternifolia*) against *H. contortus*. These findings align with those reported by Serena et al. [48], who described both in vitro and in silico anthelmintic properties of *Lannea kerstingii* and *Ficus thonningii* extracts against *Heligmosomoides polygyrus* in Cameroon. Likewise, Christelle et al. [31] reported the anthelmintic effects of ethanolic and aqueous extracts of *Khaya grandifoliola* stem bark against *H. polygyrus*, using both in vitro and in silico methods.

Our results also corroborate those of Christalin et al. [43], who demonstrated the anthelmintic activity of *Khaya anthotheca* and *Faidherbia albida* extracts, traditionally used in Chad for treating helminthiasis, along with an in silico analysis of their phytoconstituents. According to Payne et al. [49], active substances can penetrate the nematode cuticle and either disrupt postsynaptic receptors or reduce glucose uptake, resulting in larval paralysis. Gamma-aminobutyric acid (GABA) secretion, stimulated by these compounds, may further inhibit nerve impulse transmission.

Flavonoids, such as flavones, have shown anthelmintic activity [50] and induced embryonic and larval death in the free-living nematode *C. elegans* [10, 51]. Faixová et al. [52] suggest that flavonoids exert their effects by reducing sugar levels, thereby disrupting carbohydrate metabolism and depleting ATP required for essential parasite functions. Quercetin, a naturally occurring flavonoid found in onions, green tea, apples, and garlic, has also been identified as a potential neuroactive agent targeting the nervous system of *H. contortus* [53]. This implies that the anthelmintic activity observed in our extract combinations might follow similar mechanisms.

In addition, flavonol glycosides like rutin exhibit anthelmintic activity [54]. Pinocembrin (5,7-dihydroxyflavanone) has shown fasciolicide, ovicidal, and larvicidal effects on *Fasciola hepatica*, *Ascaridia galli* eggs, and *Stomoxys calcitrans* larvae, respectively [55]. Flavan-3-ols and their derivatives, such as epigallocatechin gallate, are also potent anthelmintics, capable of inhibiting egg hatching and larval development in nematodes [56].

Saponins are believed to act by inhibiting acetylcholinesterase, leading to worm paralysis and death [57]. Their adverse effects may be related to membrane disruption and increased permeability [58]. This may allow them to enter eggs and destroy their contents, thereby preventing larval development. Alternatively, they may inhibit enzymes responsible for egg hatching, reducing the hatching rate [58].

Terpenes are believed to exhibit anthelmintic activity by compromising the structural integrity of parasitic worm intestines [59]. Tannins, considered nematocidal agents, impair nutrient absorption and may bind to intestinal mucosa, leading to parasite autolysis [60]. They can also alter the nematode cuticle, reducing its flexibility and mobility [61]. Furthermore, tannins may impair the molting process by inhibiting larval sheath enzymes or disrupting associated metabolic pathways [61].

Alkaloids may exert anthelmintic effects by acting on acetylcholine receptors and inhibiting glucose uptake, ultimately resulting in parasite death [62]. Da Silva et al. [63] found that berberine and piperine are effective in vitro against nematode eggs, with berberine also being active against infective larvae. In our extracts, various secondary metabolites were identified, and bioactive compounds were successfully recovered. This confirms the findings of Usman et al. [64], who identified similar compounds in *Ficus thonningii* bark.

A good correlation was observed between the docking scores of some compounds and in vitro results. Compounds such as tricholein, isopiperolein B, pipercyclobutanamide, piperettine, and piperine from *Piper nigrum* showed very good binding affinities against SDH and also β-tubulin. This molecular interaction profile correlated well with the high in vitro activity of extracts of *P. nigrum,* especially against *H. polygyrus,* where it induced rapid larval mortality and immobility at low concentrations [65].

Similarly, the aqueous extract of *Albizia ferruginea,* which was highly active in vitro (IC_50_ = 4.729 μg/mL against *C. elegans*), had Leucokinnen IIV and Rebaudioside C compounds which were also among the best in silico binders. These compounds possessed greater predicted binding affinities than albendazole, particularly against SDH, indicating the potential for interference with mitochondrial activity in the parasites [66]. The concordance between the high docking scores of these compounds and the in vitro potency of the extract is proof of their contribution to the general anthelmintic activity [67].

Notably, although fewer docking data were reported for *Guarea cedrata*, its aqueous extract had excellent in vitro activity (IC_50_ = 3.235 μg/mL), suggesting that future computational studies should investigate its phytoconstituents more deeply. With such potent larvicidal activity, uncharacterized compounds in *G. cedrata* may have potent target binding that would be worth examining [68].

The docking results also provide plausible mechanistic explanations for the observed larvicidal activities. Inhibition of β-tubulin would destabilize microtubule polymerization, disrupting cell division and motility, while SDH targeting may impair ATP generation and oxidative phosphorylation, leading to energy depletion and worm paralysis [69]. Such mechanisms are consistent with observed motility inhibition and killing of *H. polygyrus* and *C. elegans* larvae, measured by microtracker assays.

In addition, dual-target binding molecules such as piperine and leucokinins can manifest synergistic activity by simultaneously inhibiting more than one physiological process in the parasites. Multitarget interaction is especially beneficial for minimizing the development of drug resistance and can enhance the therapeutic potential of these plant extracts [69].

To understand compound protein interactions at the molecular level, we conducted a molecular docking analysis. This helped us visualize how compounds bind at the active sites of target proteins and elucidate their biochemical mechanisms of action. The results revealed that leucokinin III, I, II, VIII, and Rebaudioside C from *Albizia ferruginea* were the most potent compounds targeting SDH and β-tubulin, outperforming the positive control (albendazole). Likewise, tricholein, isopiperolein B, pipercyclobutanamide, piperettine, and piperine from *Piper nigrum* showed high binding affinity to the same targets.

Computational studies confirmed that these compounds interact effectively with receptor proteins, primarily due to their hydroxyl and amine functional groups, as well as their flavonoid structures. The docking results strongly suggest that compounds such as tricholein, isopiperolein B, pipercyclobutanamide, piperettine, and piperine from *Piper nigrum* are promising candidates for the development of new anthelmintic agents.

## 5. Conclusion

The *P. nigrum* ethanolic extract (IC_50_ = 0.04 mg/mL) was the most active, and *G. cedrata* (IC_50_ = 3.235 μg/mL) and *A. ferruginea* (IC_50_ = 4.729 μg/mL) aqueous extracts also showed very good activity. Molecular docking supported these observations as it was seen that key compounds piperine, tricholein, and leucokinins had high binding affinities for β-tubulin and SDH, key proteins in parasite function. The congruence between docking scores and in vitro larvicidal activity provides a mechanistic basis to the observed bioactivity.

Even though the results are promising, the study limitations are that compound isolation was not done and there was no in vivo verification. The way forward should be the isolation of the most active compounds, their testing for pharmacokinetics and toxicity in animals, and verification of their mechanisms through functional assays. These results are a solid foundation for the formulation of plant-derived anthelmintic medicines and validate the traditional use of these plants for the treatment of helminth infections.

## Figures and Tables

**Table 1:** SDH inhibitor binding affinity of substances from *Albizia ferruginea*.

No.	Name of compound	Scores		Interactions			Amino acids
		XPG Score/Docking score	Glide Emodel	H-bonds	π-π bonds	Salt bridges	
1	Leucokinin III (92044001)	−8.59736	−105.029	8 (Eight)N-HS240, N-HS240, N-Ile242, N-Asn244, N-Asn244, O-Asn244, N-Thr248, Asn105-O	—	—	His240, Thr241, Ile242, Met243, Asn244, Lys247, Thr248, Trp197, Trp196, Pro193, Ser191, Asn106, Glu104, Gly103, Asn100, Pro127, Pro125, and Tyr124
2	Leucokinin I (5491285)	−7.41919	−88.6728	7 (Seven)N-Asn244, N-Asn244, N-Ile242, O-Ile242, Lys238-O, Asn198-O, Trp197-O	—	1 (One)Lys238	Ser191, Pro193, Ser194, Trp196, Trp197, Asn198, Lys238, His240, Thr241, Ile242, Met243, Asn244, Thr246, Lys247, and Thr248
3	Leucokinin VIII (134611627)	−7.34952	−90.1544	8 (Eight)N-Trp197, N-Trp197, O-Trp197, Trp197-O N-Asn198, Asn198-O, Asn214-O, and N-Asn214	2 (Two)Trp197, Trp197	2 (Two)Asp200, Asp200	Pro193, Ser194, Trp197, Asn198, Ala199, Asp200, Lys201, Lys208, Lys210, Ile212, and Asn214
4	Leucokinin II (133033)	−7.21551	−101.18	7 (Seven)N-Ile242, N-Asn244, O-Asn244, N-Asn244, Asn244-O, Trp197-O, and Lys238-O	1 (One)Trp197	—	Asn105, Glu104, Gly103, Asn100, Lys238, His240, Ile242, Met243, Asn244, Lys247, and Thr248
5	Rebaudioside C (60208888)	−6.94294	−77.4758	7 (Seven)O-Thr248, O-Gly103, O-Asn244, O-Asn244, O-Ile242, O-Thr241, and O-His240	—	—	His240, Thr241, Ile242, Met243, Asn244, Lys247, and Thr248
6	Albendazole (control positive)	−5.05772	−36.449	1 (One)N-Asn100	1 (One)Tyr124	—	Asn100, Ala102, Gly103, Lys122, Tyr124, Pro125, Leu126, Pro127, His128, Met129, and Val131

**Table 2:** SDH inhibitor binding affinity of substances from *Guarea cedrata.*

No.	Name of compound	Scores		Interactions			Amino acids
		XPG Score/Docking score	Glide Emodel	H-bonds	π-π bonds	Salt bridges	
1	Boscartol A (275376548)	−6.33722	−35.4363	1 (One)O-Thr248	—	—	Asn100, Gly103, Glu104, Asn105, Tyr124, Pro125, Pro127, His128
2	Cycloart-23-ene-3beta,25-diol (5470009)	−5.65351	−39.6324	2 (Two)O-Met129, O-Gly103	—	—	Val131, Phe130, Met129, His128, Pro127, Leu126, Pro125, Tyr124, Asn100, Gly103, Glu104, Asn105, Ala190, and Ser191
3	Boscartol G (275376549)	−5.63857	−39.2579	2 (Two)Asn244-O, and O-Gly103	—	—	Asn100, Gly103, Glu104, and Asn105
4	Melianodiol (15560457)	−5.51974	−50.9416	3 (Three)O-Gly103, O-Asn244, and His128-O	—	—	Met129, His128, Pro127, Leu126, Pro125, Tyr124, Asn100, Gly103, Glu104, Asn105, Asn244, Lys247, and Thr248
5	Scopoletin (5280460)	−5.51845	−35.2005	1 (One)O-Thr248	—	—	Asn100, Gly103, Glu104, Asn105, Thr248, Lys247, and Asn244
6	Albendazole (control positive)	−5.05772	−36.449	1 (One)N-Asn100	1 (One)Tyr124	—	Asn100, Ala102, Gly103, Lys122, Tyr124, Pro125, Leu126, Pro127, His128, Met129, and Val131

**Table 3:** Effects of the anthelminthic activities of aqueous and ethanolic plant extracts on L3 larvae of *Heligmosomoides. polygyrus bakeri*.

Plants	Extract	Concentrations (mg/mL)						IC_50_	Albendazole 400 mg	Distilled water
		0.078	0.151	0.315	0.625	1.25	2.5			
*Pipernigrum*	Aqueous	79.46 ± 0.12^**a**^	81.89 ± 0.00^**a**^	88.22 ± 0.40^**bc**^	89.65 ± 0.70^**bed**^	90.62 ± 0.21^**cef**^	91.38 ± 0.00^**df**^	0.08	100 ± 0	0
	Ethanolic	78.59 ± 0.09^**a**^	81.43 ± 0.13^**ab**^	82.48 ± 0.16^**b**^	86.22 ± 1.53	91.42 ± 0.15^**c**^	93.80 ± 0.08^**c**^	0.04	100 ± 0	0
*Albizia ferruginea*	Aqueous	39 .22 ± 0.06	47.15 ± 0.03	52.88 ± 0.80	74.35 ± 0.1	78.43 ± 00^**a**^	80.31 ± 0.0^**a**^	0.29	100 ± 0	0
*Guarea cedrata*	Aqueous	29.66 ± 1.79	50.00 ± 1.20^a^	55.75 ± 0.42^a^	67.27 ± 0.90	75.65 ± 0.62	88.31 ± 0.05	0.28	100 ± 0	0

*Note:* The lowercase letters in pairs indicating different concentrations for the same extract reflect the fact that the difference between the action of the two concentrations is not significant (*p* > 0.05), i.e., the two concentrations, although different, act in the same way on the larvae.

**Table 4:** Effects of the anthelmintic activities of aqueous and ethanolic plant extracts on L4 larvae of *Caenorhabditis elegans*.

Plants	Extract	Concentration (μg/mL)								IC_50_	Levamisole	Distilled water
		1.05	2.11	4.23	8.46	16.93	33.87	67.75	135.5			
*Albizia ferruginea*	Aqueous	10.65 ± 0.07	29.51 ± 2.80	40.64 ± 2.00	58.48 ± 0.13	74.64 ± 0.79^**a**^	74.64 ± 0.79^**a**^	86.19 ± 0.076^**b**^	95.29 ± 0.12^**b**^	4.729	100 ± 00	0
*Guarea cedrata*	Aqueous	35.50 ± 0.1200^**a**^	37.43 ± 0.04^**a**^	56.77 ± 0.07	76.38 ± 0.90^**bc**^	81.54 ± 1.60^**bd**^	81.54 ± 1.60^**cde**^	88.58 ± 2.40^**ef**^	90.29 ± 2.50^**f**^	3.235	100 ± 00	0
*Pipernigrum*	Aqueous	30.74 ± 0.01	41.43 ± 0.15	52.64 ± 0.05	64.55 ± 0.12	76.75 ± 0.06	85.71 ± 0.34	96.15 ± 0.86^**a**^	99.45 ± 0.17^**a**^	7.850	100 ± 00	0
	Ethanolic	26.48 ± 0.19	39.52 ± 0.09^**a**^	43.65 ± 0.08^**a**^	58.77 ± 0.08^**b**^	61.58 ± 0.11^**b**^	82.26 ± 0.082	97.66 ± 1.85^**c**^	98.21 ± 0.25^**c**^	16.17	100 ± 00	0

*Note:* The result is presented as Mean ± Standard Deviation. a, b, c, d, e, f for the same column, values with the same superscript are not significantly different at (*p* < 0.05).

**Table 5:** SDH inhibitor binding affinity of substances from *Piper nigrum*.

No.	Name of compound	Scores		Interactions			Amino acids
		XPG Score/Docking score	Glide Emodel	H-bonds	π-π bonds	Salt bridges	
1	Tricholein (21580214)	−6.91251	−52.5298	—	1 (One)Tyr41	—	Asn100, Gly103, Glu104, Asn105, Thr248, Lys247, Asn244, Val131, Met129, His128, Pro127, Leu126, Pro125, and Tyr124
2	Isopiperolein B (16041826)	−6.61864	−53.9157	2 (Two)His128-O, Asn105-O	1 (One)Tyr41	—	Asn100, Gly103, Glu104, Asn105, Ala190, Ser191, Thr248, Thr248, Lys247, Asn244, Val131, Met129, His128, Pro127, Leu126, Pro125, and Tyr124
3	Pipercyclobutanamide A (131751971)	−6.58554	−66.559	—	—	—	His128, Pro127, Pro125, Tyr124, Lys122, Asn100, Ala102, Gly103, Glu104, Asn105, Trp196, and Trp197
4	Piperettine (10244953)	−6.22743	−41.3268	1 (One)Asn244-O	1 (One)Tyr124	—	Val131, His128, Pro127, Pro125, Tyr124, Asn100, Gly103, Glu104, Asn105, Thr248, Lys247, Asn244
5	Piperine (638024)	−6.12209	−39.7368	1 (One)Asn105-O	—	—	Tyr124, Pro125, Leu126, Pro127, His128, Asn105, Glu104, Gly103, and Asn100
6	Albendazole (control positive)	−5.05772	−36.449	1 (One)N-Asn100	1 (One)Tyr124	—	Asn100, Ala102, Gly103, Lys122, Tyr124, Pro125, Leu126, Pro127, His128, Met129, and Val131

## Data Availability

Data are available upon request from the authors.
